# Relationship Between Dyspnoea Scales and Quality of Life in Stroke Survivors: A Retrospective Analysis

**DOI:** 10.3390/medicina61030540

**Published:** 2025-03-19

**Authors:** Abdurrahim Yildiz, Rustem Mustafaoglu, Ayse Nur Bardak

**Affiliations:** 1 Department of Physiotherapy and Rehabilitation, Faculty of Health Sciences, Sakarya University of Applied Sciences, 54400 Sakarya, Türkiye; 2 Department of Physiotherapy and Rehabilitation, Faculty of Health Sciences, Istanbul University-Cerrahpaşa, 34500 Istanbul, Türkiye; rustem.mustafaoglu@iuc.edu.tr; 3 Istanbul Physical Medicine and Rehabilitation Training and Research Hospital, Health Sciences University, 34180 Istanbul, Türkiye; abardak@gmail.com

**Keywords:** dyspnoea, scale, quality of life, respiratory muscles, stroke

## Abstract

*Background and Objectives*: The purpose of the study was to evaluate the relationship between different dyspnoea scales and clinical and physical parameters of stroke patients and to identify the most appropriate scale for stroke patients. *Materials and Methods:* This study, designed as a retrospective analysis, involved 203 patients diagnosed with stroke. Dyspnoea intensity was evaluated using four different scales: Oxygen Cost Diagram (OCD), Basic Dyspnoea Index (BDI), Modified Medical Research Council (mMRC), and Visual Analogue Scale (VAS). Respiratory muscle strength (maximal inspiratory pressure (MIP) and quality of life (Stroke Impact Scale 3.0 (SIS)) were also assessed. *Results:* The regression model explained only 20.2% of the variance in SIS total scores (R^2^ = 0.202), indicating that key predictors might be missing. Additionally, dyspnoea scales showed statistically significant but modest correlations with SIS total scores (r = 0.248–0.397), suggesting limited clinical significance. There was a statistically significant relationship between age and dyspnoea scales, except for OCD (r = −0.153, *p* = 0.056). A statistically significant relationship was found between the MIP and OCD scales (r = 0.290, *p* < 0.001) and BDI scale (r = 0.195, *p* = 0.014). However, only the BDI showed a statistically significant relationship with the other three dyspnoea scales in stroke patients. *Conclusions:* The OCD and BDI can evaluate dyspnoea ratings during day-to-day activities; therefore, these scales were significantly correlated with inspiratory muscle strength in stroke patients. Our findings suggest that while BDI and OCD are valuable tools for dyspnoea assessment in stroke patients, the overall predictive power of dyspnoea scales for quality of life is limited. Future studies should consider additional variables, such as comorbidities and rehabilitation intensity, to improve predictive accuracy and clinical relevance.

## 1. Introduction

Worldwide, stroke is the second leading cause of death and the leading cause of permanent disability [1]. A stroke affects not only the body muscles but also the respiratory system muscles [2,3]. Stroke patients often present with abnormal respiratory patterns, reduced ventilatory function, respiratory muscle weakness, and decreased diaphragmatic activity [4,5]. This abnormal respiratory function can lead to dyspnoea at high or even low exertion, which can interfere with the performance of daily activities and participation in society [6].

Dyspnoea is a common symptom affecting potentially millions of persons and can be an important symptom of cardiac, respiratory, neuromuscular, systemic, psychogenic diseases, or a combination thereof [7,8]. The physiopathology of dyspnoea is typified by a combination of factors leading to a feeling of respiratory failure. Dyspnoea occurs when pulmonary ventilation cannot meet respiratory demands. This occurs due to a disharmony between the airways, afferent receptors in the chest wall and lungs, and the respiratory motor activity of the centre. Muscle spindles on the chest wall indicate tension and rigidity in the breathing muscles. Efferent impulses are motor fiber impulses that descend to ventilatory muscles such as the diaphragm [9,10]. In the brain, central processing matches afferent and efferent signals. A discrepancy between the two signals can result in dyspnoea; for example, when the need for respiration (afferent signal) cannot be met by ventilation (efferent signal). Afferent sensory receptors enable the brain to evaluate whether motor commands to the respiratory muscles are effective. If the commands are not responded to properly, the severity of dyspnoea increases. When muscular signals are transmitted to the thoracic wall, the auditory cortex is also stimulated, and the sensation of muscle tension and dyspnoea becomes conscious [9,10,11].

Severe brain damage, such as that caused a stroke, can lead to neurogenic pulmonary oedema, which develops rapidly and can be fatal. Patients may have dyspnoea, tachycardia, hypertension, and bilateral rales [12]. In stroke patients, dyspnoea is noticed as an important symptom and can have serious effects. Forty-four percent of stroke patients reported experiencing dyspnoea after stroke, and 51% of these patients had severe dyspnoea symptoms. Eighty-five percent of stroke patients reported that dyspnoea limited their activities, and 49% reported that it prevented social participation [6]. Dyspnoea may also increase hospitalizations due to respiratory complications after stroke. These complications are among the leading causes of non-vascular deaths after stroke. Early detection and appropriate management of dyspnoea may potentially increase patients’ ability to improve their level of activity and community participation [13]. Although dyspnoea appears to be a clinically important outcome to consider during rehabilitation, its impact on any stroke population remains unknown. Several tools are currently used to evaluate dyspnoea, using a variety of instruments, such as self-report questionnaires, structured interviews, and self-report surveys, the Modified Borg Scale [14], the Visual Analogue Scale [15], the Medical Research Council Dyspnoea Scale [16], the Oxygen Cost Diagram [17], and the Baseline Dyspnoea Index [15]. Dyspnoea measurement tools have some limitations. Scales based on patients’ self-reports may be influenced by their individual perceptions, mood, and cognitive states, leading to inconsistent results. Furthermore, the lack of a universally accepted standardized measurement tool makes it difficult to make comparisons between different studies, and many scales fail to adequately assess the emotional and functional effects of dyspnoea. Some instruments may fail to detect small but clinically important changes, and external factors such as fatigue or anxiety may affect the reliability of repeated measurements [18]. However, it is not clear which of these assessment methods provides more accurate information about the severity of dyspnoea in stroke patients. Therefore, the aim of the study was to compare existing dyspnoea assessment scales and determine the most appropriate scale for stroke patients.

## 2. Materials and Methods

### 2.1. Study Design

This retrospective study was conducted by screening individuals who had a stroke and received treatment at Istanbul Physical Medicine and Rehabilitation Training and Research Hospital, Istanbul/Türkiye. Patients were included in the study from June 2016 to July 2022.

### 2.2. Participants

**Figure 1:**
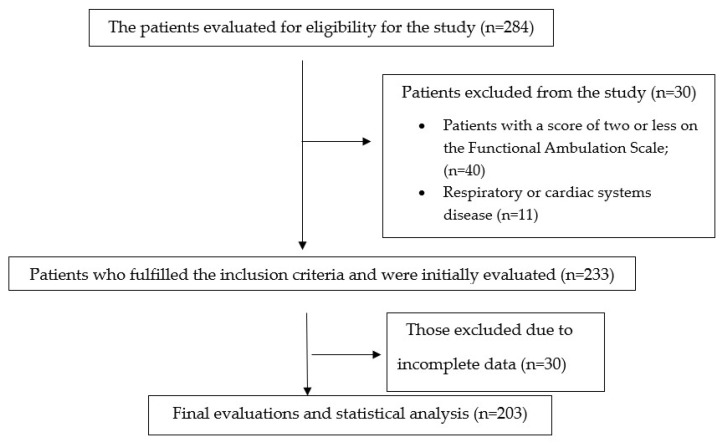
Flow chart.

The study population consisted of 203 stroke patients (119 females (58.6%)). Inclusion criteria of stroke participants are defined as follows: having a diagnosis of hemiplegia/hemiparesis; older than 18 years of age; patients with a score of two or more on the Functional Ambulation Scale; ability to understand directions; and willingness to take part in the study. Exclusion criteria: respiratory system problems such as bronchiectasis, asthma, and tuberculosis; cardiac disease; psychological treatment. The flow diagram regarding the patients included in the study is given in Figure 1.

### 2.3. Outcome Measures

#### 2.3.1. Demographic Characteristics

A patient evaluation form was used to record data of patient sex, age, body mass index (BMI), weight, height, the period since the stroke, and ethology.

#### 2.3.2. Rating of Dyspnoea

The severity of dyspnoea at rest at a given time was measured by four different clinical methods: the Oxygen Cost Diagram (OCD), the Baseline Dyspnoea Index (BDI), the Modified Medical Research Council (mMRC), and the Visual Analogue Scale (VAS). Patients were instructed in the use of these scales, with observers focusing on specific measures of dyspnoea severity within each scale.

The BDI has three main components that assess the functional effects of dyspnoea, task magnitude, and effort magnitude. Scores for each component range from 0 (very severe) to 4 (no effect), resulting in a total focal score between 0 and 12. The BDI is measured as follows: Firstly, the functional impact component assesses how much breathlessness affects the person’s daily activities. Secondly, the task magnitude component assesses which types of tasks are triggered by breathlessness. Finally, the effort magnitude component assesses the exertion level triggered by breathlessness. A rating is given for each component, and these scores are totaled to obtain the BDI focus rating. The scores are used to assess the person’s ability to cope with breathlessness and the effectiveness of interventions for this condition [19].

The OCD is a graphical analogy scale that is composed of 100 mm lines with descriptive data showing oxygen requirements at various activity levels at various points on the line. The scale ranges between 0 and 100, with a rating of 100 showing no impairment. From the lowest point of the scale to the subject’s mark, the measurement of the distance in millimetres allowed the subjects’ dyspnoea to be measured [20].

The mMRC dyspnoea scale is a tool used to measure the degree of dyspnoea in patients. This scale assesses the degree of dyspnoea experienced by patients during activities of daily living. The mMRC dyspnoea scale has five grades. Grade 0 indicates that dyspnoea is experienced only during heavy exercise. Grade 1 indicates shortness of breath when walking fast on a slope or a gentle slope. Grade 2 means walking slower than your peers on a flat road or having to stop when walking at a normal speed. Grade 3 is defined as having to stop after walking 100 m on a flat road or after walking for a few minutes. Finally, Grade 4 indicates shortness of breath when leaving the house or dressing and undressing [21].

The VAS is a simple and effective method used to assess patients’ subjective symptoms such as dyspnoea. The VAS allows patients to determine the severity of their symptoms based on their own perception and is usually applied by marking a point on a line. The VAS is a horizontal line, typically 10 cm long. One end of the line is labeled “no breathlessness” (0) and the other end is labeled “most severe breathlessness” (10). Patients mark a point on the line that represents the severity of their breathlessness. The point at which this marking is located numerically represents the severity of dyspnoea experienced by the patient [15].

#### 2.3.3. Strength of Respiratory Muscle

Breathing muscle strength was assessed using an analogue manometer (Micro RPM device, Micro Medical, Basingstoke, UK) to determine maximum inspiratory pressure (MIP). The MIP was measured and documented according to the ATS/ERS criteria [22]. The assessments were performed with the patient seated, utilizing a nose clip and a secure oral mouthpiece placed across the lips. The patients were strongly encouraged to exert maximum effort during inhalation throughout the procedure. The recorded MIP values were based on the highest measurement of at least three attempts with a maximum difference of 5 cmH_2_O between measurements. A rest pause of one minute was taken between each trial manoeuvre.

#### 2.3.4. Quality of Life

The Stroke Impact Scale (SIS) 3.0 has been utilized to evaluate stroke-related impairments and quality of life of post-stroke patients. The SIS includes eight areas: communication, strength, memory and thinking, mobility, hand function, physical and instrumental activities of daily living (i.e., ADLs and IADLs), emotion, and participation/role function. An additional question on stroke healing asks the client to rank, on a scale of 0 to 100, how much he/she feels he/she has healed after the stroke. The scores for every field range between 0 and 100, where better ratings indicate a higher quality of life [23].

### 2.4. Data Analyses

Data are presented as mean ± standard deviation (SD) and frequencies. Chi-square and independent *t*-tests were used to compare baseline values. The Pearson correlation test was performed to establish the association between variables (specifically, r = 0.5–1.0 large, 0.30–0.49 medium, and 0.1–0.29 small) [24]. Multivariate regression analysis was conducted to identify independent predictors of quality of life (SIS total score) and to evaluate the relationships between dyspnoea scales, respiratory muscle strength (MIP), and demographic factors such as age and stroke duration. Independent variables included the four dyspnoea scales (BDI, OCD, mMRC, and VAS), MIP, age, and stroke duration. The regression models were assessed for their explanatory power using R² values, and standardized beta coefficients (β) were reported to determine the relative contribution of each predictor. Statistical significance was set at *p* < 0.05, and all analyses were performed using IBM SPSS version 27.0.

## 3. Results

The total number of 203 stroke patients who participated in the study was 203, of whom 119 were female (58.6%) and 84 were male (41.4%). The primary causes of non-participation were unwillingness to participate in the study (n = 12), presence of cardiac disease (n = 7), and death (n = 3). Baseline characteristics of all subjects are reported in Table 1. The average age of the subjects was 56.51 ± 11.78 years and the average time since stroke onset was 388.39 ± 731.96 days. Most patients had an ischemic stroke (75.86%).

### 3.1. Relationship Between Age and Respiratory Muscle Strength with Dyspnoea Scales

The correlation among dyspnoea scales, age, and respiratory muscle strength is shown in Table 2. A small statistically meaningful correlation was observed between age and the dyspnoea scales, except for OCD (r = −0.153, *p* = 0.056). A statistically meaningful positive and small correlation was found between MIP and the OCD scale (r = 0.290, *p* < 0.001) and the BDI scale (r = 0.195, *p* = 0.014).

### 3.2. The Correlation Between Dyspnoea Scales and Quality of Life

There was a significant positive small to medium correlation between the OCD scale and SIS-Strength (r = 0.195, *p* = 0.028), SIS-Memory/thinking (r = 0.155, *p* = 0.083), SIS-ADL (r = 0.397, *p* < 0.001), SIS-Mobility (r = 0.319 *p* < 0.001), SIS- Hand function (r = 0.267, *p* = 0.003), SIS-Stroke Recovery (r = 0.300, *p* < 0.001), and SIS-Total (r = 0.248, *p* < 0.001). There was a significant negative small correlation between the mMRC scale and the SIS-Memory/thinking (r = −0.209, *p* = 0.019), and SIS-Total (r = −0.184, *p* = 0.039). There was a significant negative small correlation between the VAS scale and SIS-Memory/thinking (r = −0.198, *p* = 0.026), SIS-Hand function (r = −0.178, *p* = 0.046), and SIS-Total (r = −0.209, *p* = 0.019). A significant positive small correlation was found between BDI scale and SIS-Strength (r = 0.279, *p* = 0.002), SIS-Memory/thinking (r = 0.155, *p* = 0.083), SIS-ADL (r = 0.222, *p* = 0.013), SIS-Mobility (r = 0.205, *p* = 0.021), SIS-Hand function (r = 0.203, *p* = 0.023), and SIS-Total (r = 0.287, *p* < 0.001) (Table 2).

### 3.3. The Correlations Among Four Dyspnoea Scales

According to the OCD, mMRC, VAS, and BDI dyspnoea severities of the participants were 4.35 ± 2.85, 0.58 ± 0.97, 0.99 ± 1.71, and 9.50 ± 2.96, respectively. The results of our study revealed that only BDI showed a statistically significant small correlation with the other three dyspnoea scales in stroke patients (Table 3).

### 3.4. Multiple Regression Analysis

Multiple regression analysis was conducted to examine the relationships between the SIS total score, age, stroke duration, and various independent variables. In the SIS total score model, the independent variables explained 20.2% of the total variance (R^2^ = 0.202, F = 7.650, *p* < 0.001). Among these, OTD (β = 0.329, *p* < 0.001) and BDI (β = 0.188, *p* = 0.036) were identified as significant predictors of the SIS total score. In the regression model for age, the independent variables explained 9.5% of the total variance (R^2^ = 0.095, F = 4.038, *p* = 0.004). The results indicated that only BDI had a significant negative effect on age (β = −0.207, *p* = 0.014). Regarding the stroke duration model, the independent variables collectively explained only 2% of the variance (R^2^ = 0.020, F = 0.600, *p* = 0.664), and no variable was found to have a significant impact. These findings suggest that OTD is the strongest determinant of the SIS total score, while BDI exerts an inverse effect on age. However, no significant association was observed between stroke duration and the independent variables examined (Table 4).

## 4. Discussion

The present study aimed to investigate four difference scales for measuring dyspnoea in stroke patients. The clinical measures (mMRC, OCD, BDI, and VAS) are relatively simple and based on patients’ self-assessments of the difficulty of physical tasks causing dyspnoea or observer ratings of specific criteria. The findings of our study indicated a relationship between the dyspnoea scales other than OCD and age. In addition, there was a significant correlation between respiratory muscle strength and the OCD and BDI scales. However, only the BDI scale correlated with the other three dyspnoea scales in stroke patients. On the other hand, there was an association between all dyspnoea scales and quality of life.

Dyspnoea is an important frequent symptom in generalized muscle weakness. Inspiration, an active process during breathing, depends on the inspiratory muscles having sufficient strength to allow the rib cage to expand and, consequently, for air to enter the airways. This musculature may not provide sufficient chest expansion in weak patients, resulting in an inadequate inspiration, which is a major defining feature of dyspnoea [25]. Our findings provided important information in determining which scales are more effective and in which situations they should be used. Consistent with previous studies, our findings confirm once again that dyspnoea is a common and important problem in post-stroke patients. For example, similar results were reported in studies [26,27]. In particular, the negative impact of dyspnoea on activities of everyday living and social engagement has been widely documented in the literature [28]. We concluded that the dyspnoea scales found to be most effective in our study should be more widely used in the rehabilitation process of post-stroke patients. In particular, the BDI and OCD scales showed superior performance in measuring the physical and psychosocial effects of dyspnoea. According to our results, accurate and effective assessment of dyspnoea in post-stroke patients is of critical importance in rehabilitation processes. In this context, the use of the BDI and OCD scales, which were prominent in our study, can be evaluated as an important step towards increasing the patients’ quality of life. In addition, accurate dyspnoea assessment can increase patients’ participation in activities of daily living, reduce social isolation, and support general well-being.

Dyspnoea can significantly impact the health-associated quality of life of post-stroke survivors, showing correlations with ADLs and mental state [29]. Dyspnoea may influence changes in ADL performance in people with stroke; factors such as equilibrium function and walking ability play an important role in improving ADLs [30]. Deprescribing psychotropic medications, which can be associated with dyspnoea, has been linked to improvements in ADLs in post-stroke patients undergoing convalescent rehabilitation [31]. Dyspnoea can impact quality of life in older stroke patients, particularly in the areas of physical and mental health, and highlights the importance of functional independence and social support in improving their overall well-being [32]. Community-based rehabilitation by trained village health volunteers has shown positive outcomes in improving quality of life and ADLs in stroke survivors, potentially addressing issues like dyspnoea and enhancing overall well-being [33]. Stroke affects ADLs and quality of life in elderly patients, with functional independence being crucial for their overall well-being, especially in physical and mental health domains [32]. ADL interventions significantly improve cognitive function in ischemic stroke patients, enhancing their aiding recovery and quality of life. Positive quality of life impact [34].

There are many articles in the relevant literature on the effects of stroke on dyspnoea and the pulmonary system. It has been reported that stroke can affect respiratory functions and lead to dyspnoea [35]. Weakening of respiratory muscles and impairment of respiratory function can be seen after stroke, which can cause symptoms such as shortness of breath. One study found that respiratory muscle training after stroke improved the strength of inspiratory and expiratory muscles and improved lung function. It has been reported that such interventions can reduce dyspnoea and contribute to better use of breathing muscles in daily activities [36,37]. Another systematic review summarized what interventions have been used to restore pulmonary function after stroke. According to this review, respiratory muscle training, breathing exercises, and respiratory therapy were interventions that were efficacious in restoring pulmonary function in stroke patients. It also investigated the positive effects of such interventions on dyspnoea and activity [38]. In a study conducted in individuals who had a stroke, it was found that the performance of the respiratory muscles was significantly lower compared to normal individuals. This emphasizes the need to strengthen respiratory muscles after stroke [39]. Respiratory muscle training, regular physical activity, and cardiopulmonary rehabilitation programmes are effective methods to reduce dyspnoea symptoms. By improving pulmonary functions, these strategies can increase stroke patients’ quality of life [40].

The use of the BDI and OCD questionnaires in stroke patients can significantly improve the assessment of dyspnoea and its impact on health-related quality of life. These tools provide a structured approach to assess the severity of dyspnoea and its relationship to physiological impairments, which is crucial for effective management in stroke rehabilitation. Looking at the literature, the BDI has shown strong correlations with health perceptions and physical functioning in chronic obstructive pulmonary disease (COPD) patients [41]. OCD assesses the energy cost of respiration and provides insights into how dyspnoea affects daily activities. It has been found to correlate strongly with arterial blood gas abnormalities [42]. Both the BDI and OCD are interrelated and provide complementary information about dyspnoea [42] that can guide therapeutic interventions [43]. Their application in stroke patients can help identify those at risk of poor recovery due to dyspnoea and allow for targeted rehabilitation strategies. Whilst the BDI and OCD questionnaires are valuable for assessing dyspnoea, it is important to recognize that they may not capture the full extent of breathlessness experienced by stroke patients, requiring a comprehensive assessment approach that includes other clinical assessments.

This study has some limitations. Firstly, due to the retrospective design, there is the possibility of some data gaps and recording errors. This may limit our ability to fully control for potential confounding variables. Second, the sample size may not be sufficient for some subgroup analyses, which may reduce the power to detect clinical differences in more specific patient groups. Third, our study only provides short-term dyspnoea assessment. Long-term dyspnoea changes and their impact on patients’ general well-being were not assessed. Furthermore, how dyspnoea patterns change in different stroke subgroups was not analyzed in detail. Addressing these limitations in future studies will contribute to more comprehensive and generalizable results.

Our findings showed statistically significant but relatively low correlations between different dyspnoea scales and quality of life scales. This suggests that these scales are not optimal for clinical decision-making and that additional factors need to be considered to provide clinically meaningful results. The results of the regression analysis showed that the independent variables explained only 20.2% of the variance in the SIS total score. This low R² value may indicate the presence of important determinants not included in the model. Factors such as comorbidities, rehabilitation intensity, and level of functional independence could be added to the model to provide more explanatory results. Future studies should aim to create more robust predictive models by modeling with the inclusion of these variables. In addition, our findings also reveal the effect of dyspnoea on quality of life. However, the relatively low correlation coefficients indicate that more comprehensive studies are needed to create a clinically significant effect. In this context, further research should be supported by prospective cohort studies and intervention studies evaluating the long-term effects of dyspnoea symptoms.

## 5. Conclusions

Accurate and effective assessment of dyspnoea in post-stroke patients is crucial for optimizing rehabilitation outcomes. Our findings suggest that the BDI or OCD scales are more valuable tools for assessing dyspnoea in stroke patients as they correlate with respiratory muscle strength and overall dyspnoea perception. Although the BDI and OCD scales stand out as useful tools for the assessment of dyspnoea in post-stroke patients, new models that include additional factors need to be developed to use these scales more effectively in clinical decision-making. These findings may contribute to the re-assessment of dyspnoea management strategies in rehabilitation processes.

## Figures and Tables

**Table 1:** Demographic and clinical characteristics of patients.

		Total (n = 203) Mean ± SD or N	Ischemic (n = 154) Mean ± SD or N	Haemorrhagic (n = 49) Mean ± SD or N	*p*-Value
Demographic characteristics	Sex-Female/Male	119/84	93/61	26/23	0.867 ^a^
	Age (year)	56.51 ± 11.78	56.8 ± 11.9	55.7 ± 11.5	0.945 ^b^
	Height (cm)	166.81 ± 8.19	167.0 ± 8.1	166.3 ± 8.3	0.876 ^b^
	Weight (kg)	76.05 ± 13.21	74.2 ± 13.3	77.8 ± 12.9	0.945 ^b^
	BMI (kg/cm^2^)	27.27 ± 4.48	27.3 ± 4.5	26.2 ± 4.4	0.982 ^b^
	Time since stroke (day)	388.39 ± 731.96	390.2 ± 720.1	384.5 ± 750.3	0.734 ^b^
Respiratory muscle strength	MIP, cmH_2_O	45.83 ± 23.43	44.0 ± 23.5	47.2 ± 23.2	0.888 ^b^
Quality of life	SIS-Strength	59.96 ± 25.66	58.1 ± 25.7	62.4 ± 25.5	0.897 ^b^
	SIS-Memory and thinking	81.74 ± 22.07	77.0 ± 22.1	85.8 ± 22.0	0.739 ^b^
	SIS-Emotions	63.55 ± 13.71	60.7 ± 13.8	66.2 ± 13.6	0.976 ^b^
	SIS-Communication	85.25 ± 23.21	82.4 ± 23.3	88.9 ± 23.0	0.981 ^b^
	SIS-ADL	53.72 ± 21.11	54.0 ± 21.2	53.1 ± 21.0	0.911 ^b^
	SIS-Mobility	62.43 ± 27.76	64.6 ± 27.8	58.9 ± 27.5	0.671 ^b^
	SIS-Hand function	38.21 ± 21.78	40.4 ± 21.8	36.7 ± 21.6	0.564 ^b^
	SIS-Participation/role function	49.94 ± 25.13	49.2 ± 25.2	52.3 ± 25.0	0.760 ^b^
	SIS-Stroke Recovery	45.99 ± 20.44	43.1 ± 20.5	48.6 ± 20.3	0.456 ^b^
	SIS-Total	60.09 ± 14.48	57.3 ± 14.5	62.7 ± 14.4	0.345 ^b^
Rating of dyspnoea	OCD	4.35 ± 2.85	4.6 ± 2.8	4.0 ± 2.9	0.855 ^b^
	mMRC	0.58 ± 0.97	0.5 ± 1.0	0.7 ± 0.9	0.733 ^b^
	VAS	0.99 ± 1.71	1.0 ± 1.7	0.9 ± 1.7	0.921 ^b^
	BDI	9.50 ± 2.96	8.6 ± 3.0	10.3 ± 2.9	0.645 ^b^

BMI, body mass index; BDI, Baseline Dyspnoea Index; OCD, Oxygen Cost Diagram; mMRC, Modified Medical Research Council; VAS, Visual Analogue Scale; MIP, Maximum Inspiratory Pressure; SIS, Stroke Impact Scale. ^a^ Chi-square tests, ^b^ independent *t*-tests.

**Table 2:** Relationship between dyspnoea scales and age, respiratory muscle strength, and quality of life.

	OCD		mMRC		VAS		BDI	
	r	*p*	r	*p*	r	*p*	r	*p*
Age	−0.153	0.056	0.170	0.032 *	0.191	0.016 *	−0.269	<0.001 *
MIP, cmH_2_O	0.291	<0.001 *	−0.090	0.259	−0.157	0.552	0.195	0.014 *
SIS-Strength	0.195	0.028 *	−0.073	0.415	−0.110	0.218	0.279	0.002 *
SIS-Memory/thinking	0.155	0.083 *	−0.209	0.019 *	−0.198	0.026 *	0.195	0.029 *
SIS-Emotions	0.018	0.846	−0.089	0.320	−0.140	0.118	0.074	0.408
SIS-Communication	0.105	0.242	−0.092	0.303	−0.067	0.457	0.222	0.013 *
SIS-ADL	0.397	<0.001 *	−0.100	0.266	−0.123	0.169	0.205	0.021 *
SIS-Mobility	0.319	<0.001 *	−0.082	0.363	−0.107	0.233	0.101	0.261
SIS-Hand function	0.267	0.003 *	−0.157	0.079	−0.178	0.046 *	0.203	0.023 *
SIS-Participation/role function	0.132	0.140	−0.072	0.422	−0.101	0.259	0.108	0.229
SIS-Stroke Recovery	0.300	<0.001 *	−0.101	0.259	−0.101	0.260	0.109	0.224
SIS-Total	0.248	<0.001 *	−0.184	0.039 *	−0.209	0.019 *	0.287	<0.001 *

VAS, Visual Analogue Scale; mMRC, Medical Research Council Dyspnoea scale; BDI, Baseline Dyspnoea Index; OCD, Oxygen Cost Diagram; SIS, Stroke Impact Scale; ADL, Daily living activities. * *p* <0.05

**Table 3:** The relationships among the four dyspnoea scales.

	OCD		mMRC		VAS		BDI	
	r	*p*	r	*p*	r	*p*	r	*p*
OCD	-	-	−0.060	0.459	−0.021	0.791	0.238	0.003 *
mMRC	−0.060	0.459	-	-	0.821	<0.001 *	0.290	<0.001 *
VAS	−0.021	0.791	0.821	<0.001 *	-	-	0.300	<0.001 *
BDI	0.238	0.003 *	0.290	<0.001 *	0.300	<0.001 *	-	-

VAS, Visual Analogue Scale; mMRC, Medical Research Council Dyspnoea scale; BDI, Baseline Dyspnoea Index; OCD, Oxygen Cost Diagram. * *p* <0.05

**Table 4:** Multiple regression analysis between SIS total score, age, and stroke duration independent. variables and dyspnoea scales.

Predictor Variable	R^2^	Corrected R^2^	∆R^2^	∆F	∆P	Standardized β	t	*p*	Correlations		
									Zero-Order	Partial	Part
SIS total	0.449	0.175	0.202	7.650	0.001						
OCD						1.846	3.966	0.001	0.348	0.339	0.322
mMRC						1.634	0.700	0.485	−0.184	0.063	0.057
VAS						−2.040	−1.532	0.128	−0.209	−0.138	−0.124
BDI						0.805	2.118	0.036	0.287	0.189	0.172
Age	0.309	0.072	0.095	4.038	0.004						
OCD						−0.401	−1.267	0.207	−0.153	−0.102	−0.097
mMRC						−0.005	−0.003	0.998	0.170	0.000	0.000
VAS						0.845	0.937	0.350	0.191	0.076	0.072
BDI						−0.795	−20.489	0.014	−0.269	−0.197	−0.191
Duration of stroke	0.140	−0.013	0.020	0.600	0.664						
OCD						−29.784	−0.911	0.364	−0.075	−0.083	−0.082
mMRC						−141.143	−0.868	0.387	0.042	−0.079	−0.078
VAS						112.070	1.208	0.229	0.084	0.110	0.109
BDI						−3.208	−0.121	0.904	−0.041	−0.011	−0.011

VAS, Visual Analogue Scale; mMRC, Medical Research Council Dyspnoea scale; BDI, Baseline Dyspnoea Index; OCD, Oxygen Cost Diagram; SIS, Stroke Impact Scale.

## Data Availability

All authors give permission for data availability.

## Abbreviations

The following abbreviations are used in this manuscript:

ADL	Daily living activities
BDI	Baseline Dyspnoea Index
mMRC	Medical Research Council Dyspnoea scale
OCD	Oxygen Cost Diagram
SIS	Stroke Impact Scale
VAS	Visual Analogue Scale
